# Influence of Genomic Ancestry and Other Traditional Risk Factors on the Prevalence of Diabetic Peripheral Neuropathy in Admixed Individuals With Type 1 Diabetes in Brazil: A Pioneer Multicenter Study

**DOI:** 10.1111/jns.70049

**Published:** 2025-08-13

**Authors:** Cejana Hamu Aguiar, Hermelinda Cordeiro Pedrosa, Lucianne Righeti Monteiro Tannus, Livia Leite Ferreira, Dayse Silva, Luís Cristóvão Porto, Carlos Antonio Negrato, Marilia Brito Gomes

**Affiliations:** ^1^ Research Center of Foundation for Education and Research in Health Sciences Endocrinology Unit of the Regional Hospital of Taguatinga, Secretariat of Health of the Federal District Brasília Brazil; ^2^ Department of Internal Medicine Diabetes Unit, State University of Rio de Janeiro Rio de Janeiro Brazil; ^3^ DNA Diagnostic Laboratory Rio de Janeiro State University Rio de Janeiro Brazil; ^4^ Histocompatibility and Cryopreservation Laboratory (HLA) Rio de Janeiro State University (UERJ) Rio de Janeiro Brazil; ^5^ University of Sao Paulo, Faculty of Medicine of Bauru Bauru Brazil

**Keywords:** diabetic peripheral neuropathy, genomic ancestry, self‐reported color‐race, type 1 diabetes

## Abstract

**Aims:**

To assess the influence of genomic ancestry (GA) and other traditional risk factors on the prevalence of diabetic peripheral neuropathy (DPN) in admixed Brazilian individuals with type 1 diabetes (T1D).

**Methods:**

This cross‐sectional, multicenter, pioneer study was conducted in 14 public clinics in 10 Brazilian cities. From 1760 individuals, 1732 were included (98.4%), aged 29.9 ± 11.9 years, diabetes duration 15.4 ± 9.2 years, 968 females (55.9%), 939 (55.7%) self‐reported as White. DPN was evaluated by the validated neuropathy disability score (NDS) and neuropathy symptoms score (NSS).

**Results:**

The prevalence of DPN was 14.8%. In hierarchical multivariate logistic regression, the covariates associated with DPN were age, diabetes duration, HbA1c, type of health care insurance, insulin therapeutic regimen, number of yearly clinical visits, low exercise practice rates, hypertension, dyslipidemia, heart rate, statin use, uric acid levels, lower health‐related quality of life, presence of diabetic retinopathy, and amputations. Among the sociodemographic characteristics, African GA, a contemporary emerging factor, showed the highest association.

**Conclusions:**

DPN was related to several comorbidities, diabetes‐related complications, and lower health‐related quality of life. These individuals were young, implying a high lifetime cost of this disease. The association with the emerging factor African GA warrants further studies involving other admixed populations.

**Abbreviations**
ASCVD: atherosclerotic cardiovascular disease
BMI: body mass index
BrazDiab1SG: Brazilian Type 1 Diabetes Study Group
CAN: cardiovascular autonomic neuropathy
CSII: continuous subcutaneous insulin infusion
CVD: cardiovascular disease
CVRF: cardiovascular risk factors
DAN: diabetic autonomic neuropathies
DKD: diabetes kidney disease
DN: diabetic neuropathies
DPN: diabetic peripheral neuropathy
DR: diabetic retinopathy
DSPN: distal symmetric polyneuropathy
eGFR: estimated glomerular filtration rate
GA: genomic ancestry
HbA1c: glycated hemoglobin
HCI: health care insurance
HR: heart rate
HRQoL: health‐related quality of life
IBGE: Brazilian Institute of Geography and Statistics
ITR: insulin therapeutic regimens
NDS: neuropathy disability score
NSS: neuropathy symptoms score
SDoH: social determinants of health
SMBG: self‐monitoring blood glucose
T1D: type 1 diabetes
UACR: urinary albumin‐to‐creatinine ratio

## 1 Introduction

Diabetic neuropathies (DN) are the most common diabetes‐related chronic complications, and their estimated lifetime prevalence surpasses 50% among individuals with type 1 diabetes (T1D) [1]. Recent data have demonstrated that it also can occur in young individuals with T1D [2], those with undiagnosed diabetes and/or prediabetes [3], metabolic syndrome, and obesity [4, 5]. Among the various forms of DN, diabetic peripheral neuropathy (DPN) and diabetic autonomic neuropathies (DAN), particularly cardiovascular autonomic neuropathy (CAN), are unequivocally the most studied [1, 6]. Moreover, recent data highlight the impact of other forms of DAN, such as gastrointestinal and urogenital autonomic neuropathies [1].

Consensually, DPN is defined as a symmetrical, length‐dependent sensorimotor polyneuropathy associated with metabolic and microvascular alterations resulting from chronic hyperglycemia and the presence of cardiovascular risk factors (CVRF) [7]. A European study by Tesfaye et al. [8] showed for the very first time, 20 years ago, an existing link between CVRF and DPN in individuals with T1D [8]. More recently, other emerging risk factors have been related to DPN, such as social determinants of health (SDoH) [9].

Neuropathy may occur early in individuals with T1D, and previous data collected among children and adolescents have demonstrated a wide variation in DN prevalence of up to 60%, reflecting the diverse screening and diagnostic methods used in different studies, with different sensitivities and specificities [10].

There are limited data evaluating DPN in Brazilian individuals with T1D, generally analyzing local or regional samples which are not representative of the total Brazilian population. The Brazilian Type 1 Diabetes Study Group (BrazDiab1SG) collected data regarding different aspects of T1D, including the prevalence of CAN [11].

On the other hand, self‐reported color‐race, applied in national population surveys, including the BrazDiab1SG, could lead to misclassifications. This might happen due to the high miscegenation rates found among the three ancestral roots that formed the Brazilian population: the Native Amerindians, Europeans, and Sub‐Saharan Africans, resulting in one of the most admixed populations in the world as described in a multicenter Brazilian study comprising individuals with T1D and controls [12]. Consequently, the inclusion of genomic ancestry (GA) along with self‐reported color‐race could shed some light to identify and understand the possible racial and ethnic disparities in the prevalence of DPN in our country. It should be noted that self‐reported color‐race is a subjective construct influenced by cultural and regional contexts that might obscure true genetic disparities and compromise the accuracy of risk group identification. Otherwise, a recent Brazilian national study evaluating the whole genome sequence data of healthy individuals [13] has established that the large admixture found in the Brazilian population is completely different from other South American countries regarding the proportion of European, African, and Native Amerindian GA [14]. This means that studies aiming to establish the connection between some diseases and GA are extremely important.

Therefore, this multicenter pioneer study aimed to find the influence of GA, as an emerging factor, and other traditional risk factors on the prevalence of DPN in a national cohort of admixed individuals with T1D in Brazil, involving all the geographical regions of the country.

## 2 Participants and Methods

This was a cross‐sectional, national multicenter study conducted between August 2011 and August 2014 in 14 public clinics, located in 10 Brazilian cities, from all five geographic regions. Research design and methods have been previously detailed [15]. Briefly, each clinic provided data from at least 50 individuals with T1D receiving healthcare from the Brazilian Unified Health System (SUS). Our sample included 1760 individuals with T1D diagnosed between 1960 and 2014. This study was approved by all study centers ethics committees.

Participants were included if they were 13 years or older, had a minimum 6 months medical follow‐up at the respective center. T1D diagnosis was made by a physician and based on the classical clinical presentation (polyuria, weight loss, polydipsia, and continuous insulin use since then). Those who did not fulfill the inclusion criteria were excluded (pregnant or lactating women, an acute infection presence or ketoacidosis in the previous 3 months of enrollment). Written informed and free consent was obtained from all participants and/or from their parents or responsible person, if necessary.

The following variables were assessed: current age, age at diagnosis, self‐reported color‐race White, Black, Brown (“parda”), Asian (“amarela”), and Indigenous (“indígena”) [16], diabetes duration, school attendance years, smoking status, prescribed type of insulin therapeutic regimens (ITR) and body mass index (BMI). ITR were stratified as follows: intermediate insulin (NPH) or long‐acting insulin analogs only; NPH plus regular insulin or long‐acting insulin analogs plus short‐acting insulin or regular insulin; or use of continuous subcutaneous insulin infusion (CSII).

Health care insurance (HCI) was defined as only public or public plus private. We considered exercise practice as a self‐reported frequency of physical activities at least three times a week. Hypertension was defined by the exclusive anti‐hypertensive drugs use for its treatment. Dyslipidemia was confirmed by abnormal lipid levels according to the American Diabetes Association or elevated LDL based on atherosclerotic cardiovascular [ASCVD] risk factors or statins use [17]. CVD included conditions such as coronary artery disease (CAD), peripheral vascular disease (PVD), or cerebrovascular disease. Good glycemic control was defined as HbA1c < 7.0% [17, 18]. All the above measurements were performed in a single center (State University of Rio de Janeiro). Health‐related quality of life (HRQoL) was evaluated by EQ‐VAS a part of EQ‐5D, that is based on general self‐rated health and also comprises of a visual scale of general health status ranging from 0 to 100 [19].

For diabetes kidney disease (DKD), the presence of microalbuminuria (urinary albumin‐to‐creatinine ratio [UACR] 30 to < 300 mg/g) or macroalbuminuria (UACR ≥ 300 mg/g) and/or estimated glomerular filtration rate (eGFR) < 60 mL/min/1.73 m^2^, measured by Chronic Kidney Disease Epidemiology Collaboration (CKD‐EPI) in adults as stated by KDIGO [20] and application of the adolescent Schwartz formula [21] were the definition criteria used.

Diabetic retinopathy (DR) was evaluated by mydriatic binocular indirect ophthalmoscopy on the CANON 30‐2 Shimomaruko 3‐Chome retinograph (Ohta‐KU, Tokyo, Japan) with the ImageNet Lite system. The diagnosis was based on the eye with the highest level of commitment as: absent, non‐proliferative DR, proliferative DR, and macular edema, according to the DR international classification [22].

Each center and staff were trained for the DPN evaluation, following a protocol based on the original Neuropathy Disability Score (NDS) and Neuropathy Symptom Score (NSS). Both scores were translated and adapted into Brazilian Portuguese, demonstrating good reliability (Cronbach's' alpha *r* = 0.76, *p* < 0.0001). DPN diagnosis, using the combination of NDS‐ECN (Escore de ComprometimentoNeuropático) and NSS‐ESN (Escore de SintomasNeuropáticos) showed also a good reliability (*r* = 0.63, *p* < 0.0001) [23].

Neuropathic deficits in the feet, related to small fibers, were identified by testing for the pinprick sensation (unmyelinated Type C fiber, using a wooden toothpick) and temperature (thinly myelinated A delta (δ) fiber, using the 128 Hz tuning fork cool tines). Large fiber deficits were assessed by testing vibration (myelinated A beta (β), with a 128 Hz tuning fork) and Achilles reflex (myelinated A alpha (α), using a Babinski hammer). NDS scores were assigned as follows: 1 for reduced or absent sensation in each foot, and 0 if the sensation was present. For reflexes, scores were 2 if the reflex was absent, 1 if present only upon reinforcement, and 0 if present. The NDS total score ranged from 0 to 10, with classification as follows: 0–2 normal, 3–5 light, 6–8 moderate, and 9–10 severe [23, 24, 25].

Participants were asked about feeling pain or discomfort in the feet and/or legs to register the NSS. Burning, numbness, or tingling scored 2, while fatigue, cramping, or aching scored 1. The presence of symptoms was scored as 2 for the feet, 1 for the calves, and 0 for other sites. Pain or discomfort exacerbation was scored 2 for night, 1 for day and night, and 0 for daytime only, with an additional score of 1 if the participant had woken by symptoms. Maneuvers to reduce symptoms were scored 2 for walking, 1 for standing, and 0 for sitting or lying down. The maximum NSS was 9, with severity graded as: 0–2 none, 2–4 mild, 5–6 moderate, and 6–9 severe. DPN was defined by moderate symptoms and mild neurologic signs (NSS ≥ 5 and NDS ≥ 3), or DPN without neuropathic pain if NDS ≥ 6 [23, 24, 25].

The study sample calculation has been presented previously [15]. Shortly, it represented the T1D case distribution in geographic regions of Brazil, and it was calculated by the 2000 Brazilian Institute of Geography and Statistics Population Census (IBGE) at the time the BrazDiab study was launched [26]. The national diabetes prevalence data were applied to determine the minimum individual number to be studied in each region [27].

Socioeconomic status was defined according to the Brazilian Economic Classification Criteria [28] and the following categories were considered for this analysis: high, middle, low, and very low. This classification also accounts for education level: illiterate/incomplete primary education, complete primary education/incomplete secondary education, complete secondary education/incomplete high school, complete high school/some college, or college graduate [29].

Genomic DNA was extracted from peripheral blood with the commercial kit SP QIA symphony by automation with QIA symphony equipment, following the manufacturer's instructions (Qiagen, USA). The global and individual GA were inferred using a panel of 46 AIM‐INDEL [30]. Genotyping was done by multiplex PCR and capillary electrophoresis with ABI 3500 sequencer. Allele naming was performed with the software Gene Mapper V.4.1 (Life Technologies, USA). Estimated ancestry was assessed with the Structure V.2.3.3 software and the HGDP‐CEHP diversity panel (Sub‐Set H952) as reference ancestral population data.

## 3 Statistical Analysis

Exploratory analysis was performed to assess clinical, demographic, and laboratory data stratified according to the presence of DPN. Both analyses were presented as means (± SD) or median, interquartile range [IQR] for continuous variables and as counts (relative frequencies) for discrete variables. Parametric *Student's t*‐*test* and nonparametric Mann–Whitney test were used for comparison between groups as indicated.

For DPN relationship with demographic, laboratorial data, diabetes management, diabetes‐related chronic complications, severe or asymptomatic hypoglycemia, and other traditional‐related DPN risk factors, hierarchical multivariate logistic regression was conducted using five levels (sociodemographic, diabetes management and treatment, clinical data, laboratorial data and diabetes‐related complications) to determine their association with DPN. In addition, each level was adjusted for the covariates present in the previous model. Model 1 was adjusted for age, gender, diabetes duration, socioeconomic status, type of HCI, smoker, alcoholism, years of school attendance, and self‐reported color‐race or GA (both were tested separately). Model 2 was adjusted for HbA1c levels, ITR, number of clinical appointments/years, level of care, time of follow‐up in each center, and exercise practice. Model 3 was adjusted by the presence of hypertension, dyslipidemia, heart rate (HR), use of statins, and anti‐hypertensive drugs. Model 4 was adjusted for the levels of triglycerides, C‐reactive protein, and uric acid. Model 5 was adjusted for the presence of any hypoglycemia and the presence of severe hypoglycemia, eGFR, presence of DR, DAN‐CAN, DKD, and any amputation.

To select the independent variables, we chose those with statistical significance in an exploratory analysis (*p* < 0.2) or those with clinical plausibility. The model fit was assessed through a Hosmer and Lemeshow test and Omnibus test. The calculated Nagelkerke *R* ^2^ and the odds ratio (OR) with a 95% confidence interval (CI) were expressed as indicated. A two‐sided *p* value < 0.05 was considered statistically significant. A statistical analysis was performed using SPSS Statistical Package for the Social Sciences (SPSS) version 17.0, SPSS Inc., Chicago, Illinois, USA.

## 4 Results

### 4.1 Overview of Demographic Data of the Studied Population

Overall, data were obtained from the 1732 (98.4%) participants. Demographic and socioeconomic data of the studied population are described in Table 1.

**TABLE 1:** Clinical, socioeconomic and demographic data of the individual studied.

Demographic data	
*N*	1732
Gender, female *n* (%)	968 (55.7)
Age, years	29.9 ± 11.9
Age at diagnosis, years	14.7 ± 8.9
Diabetes duration, years	15.4 ± 9.2
Time of follow‐up, years	7.9 [9.0]
Years of school attendance (years)	12.2 ± 3.8
Self‐reported color‐race, *n* (%)	
White	939 (54.2)
Black	134 (7.7)
Brown	624 (36.0)
Asian	20 (1.1)
Indigineous	16 (0.9)
Socioeconomic status, *n* (%)	
High	53 (3.1)
Medium	785 (45.3)
Low	837 (48.3)
Very low	57 (3.3)
Level of care, *n* (%)	
Secondary	650 (37.5)
Tertiary	1082 (62.5)
Health care insurance, *n* (%)	
Only public	1209 (69.8)
Private plus public	523 (30.2)
Data related to diabetes treatment and glycemic control	
HbA1c (%)	9.0 ± 2.1
Insulin therapeutic regimens, *n* (%)	
Intermediate or long acting	86 (5.0)
Intermediate or long acting plus short acting or regular	1584 (91.5)
Continuous subcutaneous insulin infusion	62 (3.6)
Insulin (IU/kg)	0.86 ± 0.38
Smoker, yes, *n* (%)	91 (5.3)
Alcohol consumption, yes, *n* (%)	160 (9.8)
Exercise practice, yes *n* (%)	897 (51.9)

*Note:* Data are presented as *n* (%), mean ± SD or median [IQR, interquartile range].

### 4.2 Overview of Demographic, Ethnicity (Self‐Reported Color‐Race and Genomic Ancestry) Clinical and Laboratory Data of the Studied Population Stratified According to the Presence of DPN

The prevalence of DPN in the studied population was 14.9% (*n* = 262). Clinical, socioeconomic, demographic, and laboratory data of the studied population according to the presence of DPN are listed in Table 2. DPN was associated with older ages, diabetes duration, time of follow‐up in each center, number of yearly clinical appointments, lower socioeconomic status, lower years of school attendance, and lower HRQoL. Self‐reported color‐race was not related to the presence of DPN, but African GA showed a tendency. Participants with DPN were attended more frequently in tertiary care centers and had less HCI (public and private).

**TABLE 2:** Clinical, demographic and laboratory data stratified by the presence of peripheric neuropathy.

*N* total	1732		*p*
	No	Yes	
Number (%)	1470 (83.5)	262 (14.9)	
Demographic data			
Gender, female *n* (%)	823 (56.0)	144 (55.2)	0.7
Age, years	28.6 ± 11.4	37.6 ± 12.1	< 0.001
Diabetes duration, years	14.3 ± 8.6	21.7 ± 9.9	< 0.001
Age at diagnosis, years	14.4 ± 8.9	16.9 ± 8.7	0.008
Time of follow up, years	7.25 [8.0]	11.0 [14.25]	< 0.001
Level of care, tertiary *n* (%)	901 (61.3)	181 (69.3)	0.013
Health care insurance (public and private), yes *n* (%)	469 (31.9)	54 (20.6)	< 0.001
Years of study, years	12.3 ± 3.7	11.6 ± 3.9	0.007
Smoker, yes *n* (%)	72 (4.9)	19 (7.3)	0.13
Alcoholism, yes *n* (%)	137 (9.3)	23 (8.8)	0.8
Ethnicity, *n* (%)			
**Self‐reported color‐race, yes *n* (%)**			0.7
White	796 (54.1)	143 (54.4)	
Black	111(7.5)	23 (8.8)	
Brown	533 (36.2)	91 (34.9)	
Asian	16 (1.0)	4 (1.5)	
Indigineous	15 (1.0)	1 (0.4)	
Genomic ancestry (%)			
European (%)	66.6 ± 20.3	64.8 ± 21.8	0.2
African (%)	19.7 ± 16.4	21.7 ± 18.7	0.1
Native Amerindian (%)	13.6 ± 11.3	13.5 ± 11.9	0.9
Socioeconomic status (%)			0.02
High	48 (3.3)	5 (1.9)	
Medium	679 (46.2)	107 (40.8)	
Low	699 (47.5)	138 (52.9)	
Very low	45 (3.1)	12 (4.6)	
Diabetes management and treatment			
HbA1c (%)	8.9 ± 2.1	9.2 ± 2.4	0.078
HbA1c (mmol/mol)	74.6 ± 22.8	77.3 ± 25.8	
HbA1c (%) year before	8.9 ± 2.0	9.3 ± 2.5	0.02
HbA1c (mmol/mol), year before	74.1 ± 24.1	78.3 ± 28.2	
Insulin dose (U/kg/day)	0.86 ± 0.36	0.86 ± 0.44	0.9
SMBG^a^, yes *n* (%)	1391 (94.6)	244 (93.1)	0.4
SMBG^a^, *n*	3.6 ± 1.7	3.7 ± 1.4	0.5
Adherence to diet, yes *n* (%)	752 (58.2)	132 (57.1)	0.7
Physical activity, yes *n* (%)	790 (53.8)	107 (40.8)	< 0.001
Number of clinical appointments/years	3.6 ± 1.7	4.0 ± 1.9	0.001
Insulin therapeutic regimens, *n* (%)			0.01
Intermediate or long acting	67 (4.6%)	19 (7.3)	
Intermediate or long acting plus short acting or regular	1345 (91.4)	240 (91.6)	
Continuous subcutaneous insulin infusion	58 (3.9%)	3 (1.1)	
Bolus (%)	0.33 ± 0.14	0.32 ± 0.15	0.2
Clinical data			
BMI (kg/m^2^)	24.1 ± 4.1	24.4 ± 4.4	0.3
Systolic blood pressure	120.8 ± 15.0	128.2 ± 20.2	< 0.001
Diastolic blood pressure	74.7 ± 10.1	77.8 ± 11.8	< 0.001
Hypertension, yes *n* (%)	213 (14.5)	96 (36.6)	< 0.001
Dyslipidemia, yes *n* (%)	261 (17.8)	112 (42.7)	< 0.001
Heart rate	78.2 ± 12.3	81.5 ± 13.8	< 0.001
Laboratory data			
Triglycerides (mg/dL)	84 [59.0]	98 [66.0]	0.02
HDL‐cholesterol (mg/dL)	56.7 ± 18.6	55.5 ± 20.8	0.3
Uric acid (mg/dL)	4.9 ± 1.7	5.8 ± 2.3	< 0.001
Total cholesterol (mg/dL)	188.2 ± 52.6	188.5 ± 45.0	0.9
LDL‐cholesterol (mg/dL)	110.4 ± 41.1	110.4 ± 35.7	0.9
Non‐HDL‐cholesterol (mg/dL)	131.0 ± 47.9	132.9 ± 44.8	0.6
Creatinine (mg/dL)	1.00 [0.32]	1.1 [0.48]	< 0.001
C reactive protein (mg/dL)	0.16 [0.41]	0.24 [0.61]	0.002
TSH mIU/L	2.1 [1.8]	2.2 [2.0]	0.2
Free T4 ng/dL	1.29 ± 0.29	1.27 ± 0.26	0.2
B12 vitamin (pg/mL)	547.9 ± 242.9	548.3 ± 280.8	0.9
Medications			
Anti‐hypertensive drugs, yes *n* (%)	342 (23.4)	124 (48.1)	< 0.001
Statin yes *n* (%)	257 (17.5)	119 (45.4)	< 0.001
HRQoL^b^ and diabetes‐related chronic complications			
HRQoL^b^	72.6 ± 16.7	64.5 ± 19.7	< 0.001
Any hypoglycemia, yes *n* (%)	1087 (73.9)	208 (79.7)	0.047
Severe hypoglycemia, yes *n* (%)	198 (18.2)	48 (23.1)	0.10
Asymptomatic hypoglycemia	408 (37.5)	86 (41.3)	0.3
Cardiac autonomic neuropathy, yes *n* (%)	280 (19.4)	107 (42.7)	< 0.001
Retinopathy, yes *n* (%)	431 (30)	167 (67.1)	< 0.001
DKD^c^, yes *n* (%)	321 (30.1)	117 (60.9)	< 0.001
Glomerular filtration rate (mL/min/1.73 m^2^)	89.5 ± 29.4	72.5 ± 30.2	< 0.001
Albuminuria (mg/dL)	8.4 [16.9]	16.0 [80.5]	0.005
Macrovascular disease, yes *n* (%)	25 (1.7%)	13 (5.0%)	0.001
Any amputation, yes *n* (%)	5 (0.3)	14 (5.3)	< 0.001

*Note:* Data are presented as *n* (%), mean ± SD or median [IQR, interquartile range].

^a^ SMBG, self‐monitoring blood glucose.

^b^ HRQoL, health‐related quality of life.

^c^ DKD, diabetes kidney disease.

Individuals with DPN had a tendency to have higher current HbA1c and also higher levels of HbA1c performed in the previous year. These individuals practiced physical activities less frequently and had a higher prevalence of any type of hypoglycemia without a difference in the prevalence of severe and asymptomatic hypoglycemia.

Participants with DPN presented higher systolic blood pressure, diastolic blood pressure, HR, prevalence of hypertension and dyslipidemia, use of statins, and use of renin‐angiotensin system inhibitors. Higher levels of uric acid, creatinine, C‐reactive protein, and albuminuria and a lower eGFR (mL/min/1.73 m^2^) were noted among individuals with DPN; no difference was noted concerning the levels of total cholesterol, non‐HDL‐cholesterol, LDL‐cholesterol, and triglycerides. Referring to the other diabetes‐related complications, participants with DPN also had a higher prevalence of DR, DKD, DAN‐CAN, and macrovascular disease. Overall, very few participants (19, 1.1%) presented amputation; those who had limb loss 16 (84.2%) were below the ankle (minor) and three (15.8%) above the ankle (major). Of these amputations, seven (36.8%) were of both limbs.

### 4.3 Hierarchical Multivariate Analysis With DPN as Dependent Variable (Outcome)

The hierarchical logistic regression analysis data are described in Table 3. Model 1 showed that age, diabetes duration, years of school attendance, HCI (public plus private) and African GA were associated with the presence of DPN. In model 2, the level of HbA1c, ITR (use of intermediate or long‐acting insulin) were also associated with DPN; meanwhile, the practice of exercise was negatively associated with DPN. In model 3, hypertension, dyslipidemia, HR, and the use of statins were associated with DPN as well. In model 4, uric acid was associated with DPN; and in model 5, the presence of any DR and any type of amputation showed an association with DPN. In this model, a tendency for an association was observed with the presence of DAN, and a negative association was noted with HRQoL.

**TABLE 3:** Hierarchical multiple logistic regression analysis of sociodemographic, diabetes management, clinical, laboratory and diabetes‐related complications data associated with diabetic peripheral neuropathy(outcome).

Factors	Adjusted OR	95% CI	*p*
Sociodemographic level (Model 1)			
Female (Ref: Male)	1.042	0.8–1.358	0.758
Age, years	1.060	1.049–1.072	< 0.001
Duration of diabetes, years	1.052	1.033–1.071	< 0.001
Years of school attendance, years	0.948	0.914–0.983	0.004
Social economic status (Ref: very low)			
High	0.415	0.119–1.451	0.168
Medium	0.601	0.281–1.284	0.189
Low	0.822	0.398–1.700	0.598
Health insurance (Ref: public and private)	0.650	0.455–0.929	0.018
Smoker, yes	1.340	0.766–2.345	0.305
Alcoholism, yes	1.236	0.752–2.028	0.402
African genomic ancestry^a^	2.304	1.016–5.225	0.046
Diabetes management and treatment level (Model 2)			
HbA1c (%)	1.131	1.055–1.213	0.001
Insulin regimens (Ref: CSII)			
Intermediate or long acting	4.798	1.183–19.451	0.028
Intermediate or long acting plus short acting or regular	3.244	0.925–11.375	0.066
Number of clinical appointments/years	1.117	1.027–1.216	0.01
Level of care (Ref: Tertiary)	0.788	0.576–1.077	0.135
Time of follow‐up, years	0.991	0.975–1.010	0.374
Exercise practice, yes	0.607	0.450–0.819	0.001
Clinical data (Model 3)			
Hypertension, yes	1.560	1.101–2.211	0.012
Dyslipidemia, yes	2.003	1.434–2.797	< 0.001
Heart rate	1.022	1.010–1.035	< 0.001
Statin use, yes	1.699	1.116–2.646	0.014
Anti‐hypertensive drugs use, yes	1.121	0.752–1.670	0.576
Laboratory data (Model 4)			
Triglycerides (mg/dL)	1.000	0.99–1.002	0.703
C‐reactive protein (mg/dL)	1.068	0.955–1.196	0.24
Uric acid (mg/dL)	1.162	1.063–1.271	0.001
Diabetes complications and health quality of life level (Model 5)			
Health‐related quality of life	0.983	0.975–0.992	< 0.001
Any hypoglycemia, yes	1.371	0.931–2.018	0.11
Severe hypoglycemia, yes	1.037	0.675–1.595	0.868
Retinopathy, yes	2.046	1.432–2.922	< 0.001
eGlomerular filtration rate, mL/min/1.73 m^2^	0.999	0.992–1.006	0.74
Diabetic autonomic neuropathy, yes	1.388	0.951–2.025	0.090
Diabetes kidney disease, yes	1.488	0.926–2.392	0.10
Amputation, yes	17.873	2.168–147.32	0.007

*Note: p* < 0.05.

Abbreviations: 95% CI = 95% confidence interval; OR = Odds ratio; Ref = Reference category.

^a^ The use of self‐reported color‐race instead of African Genomic ancestry did not reach statistical significance Each level was adjusted for the covariates present in the previous model.

Considering the adjusted final model, all clinical and laboratory independent variables that were part of the model could explain 33.9% (Nagelkerke R‐squared) of a given participant having DPN. Overall, 87.3% of the participants were correctly classified by the model.

## 5 Discussion

To the best of our knowledge, this was the first multicenter study conducted in South America, a region characterized by huge differences in genetic background among the countries. This study evaluated specifically the prevalence of DPN in T1D Brazilian individuals, which was found to be approximately 15% (14.9%). The most important factors associated with DPN were age, diabetes duration, HbA1c levels, hypertension, dyslipidemia, HR, statins use, uric acid levels, lower HRQoL, presence of DR, and amputations.

Besides the traditional risk factors mentioned above patients with DPN had more yearly clinical visits, less HCI, more frequently NPH and long‐acting insulin use alone, lower exercise practice rates, and increased African GA. In addition, these young patients had lower HRQoL that negatively impacted their lives in the most productive years.

Differences in the prevalence of DPN are found worldwide and have been attributed to several factors due to different diagnostic criteria and definitions, and also to sociodemographic, clinical, and laboratorial data of the studied populations, including their genetic background [2, 8, 31, 32]. The ~15% prevalence of DPN found in our study is similar to the 17.5% (95% CI 4.8%–30.2%) figure found in a systematic review and meta‐analysis which evaluated individuals with T1D [31]. In this review, as expected, differences in diagnostic criteria had an important impact on the wide variation range of the prevalences [1, 7].

A multicenter study conducted in Beijing, including 73 individuals with T1D, found a DPN prevalence of 21.92% [33]. Another Chinese study, the China Diabetes Type 1 Study (CD1S), revealed that 45.3% of hospitalized individuals with T1D had DPN [34]. The studies conducted by Mizokami‐Stout et al. in the United States and by Jeyam et al. in Scotland, with representative population samples, have found 11% and 13% of DPN in patients with T1D, respectively [2, 9], more similar to the one found in our study.

The prevalence of DPN among individuals with T1D in South America is not completely documented in the currently available studies. A systematic review and meta‐analysis on the prevalence of DPN in Latin America and the Caribbean, which included data from several countries from this region, did not differentiate DPN based on T1D and type 2 diabetes, reporting an overall 46.5% diabetes prevalence [35].

In the present Brazilian cohort of individuals with T1D, traditional risk factors for DPN, by multivariate hierarchical analysis, were older age, longer diabetes duration, more yearly clinical visits, less HCI and school attendance years, exclusive use of NPH or long‐acting insulins, and exercise practice lower rates. In addition, these young individuals had also lower HRQoL, higher HbA1c levels, HR, greater frequency of hypertension, dyslipidemia, statins use, DR, and amputations. The majority of the above risk factors have been described in other epidemiological studies conducted in individuals with T1D [2, 8]. Moreover, no association was found with female gender, as has been observed in EURODIAB [8] and, surprisingly, in triglycerides levels in comparison to individuals with T1D enrolled in the Search [2] and EURODIAB [8] studies, probably due to the lower BMI observed in our participants.

In our study, individuals with DPN reported lower rates of exercise practice, a trend also observed in previous studies. The literature suggests that regular physical activity may reduce the risk of neuropathy, possibly by improving insulin sensitivity, endothelial function, and reducing systemic inflammation [1, 36, 37]. Encouraging exercise as part of diabetes management may, therefore, offer additional benefits in mitigating neuropathy risk.

Among the emerging risk factors, we have found that higher rates of African GA and levels of serum uric acid were found in individuals with DPN. The former risk factor has been described in all studies evaluating diabetes‐related complications in the BrazDiab1SG, mostly regarding DKD [38], retinopathy [39] and CAN [11].

In a previous analysis of data from the BrazDiab1SG, we found an inverse correlation between serum uric acid levels and eGFR, with a decrease of 4.11 mL/min/1.73 m^2^ in eGFR for every 1 mg/dL increase in serum uric acid levels [40]. The possible mechanisms that could explain the association among diabetes‐related chronic complications and serum uric acid levels include its pro‐inflammatory, pro‐oxidant effects and endothelial function, which can cause endothelial damage and dysfunction [41].

On the other hand, the literature suggests that in type 2 diabetes, elevated serum uric acid levels are associated with worse nerve function, as demonstrated in studies correlating serum uric acid levels with the severity of diabetic sensorimotor neuropathy [42]. These findings might be relevant for understanding the role of serum uric acid in T1D; although more research is needed to establish a direct relationship with DPN. Although there is evidence that serum uric acid may be associated with vascular dysfunctions in T1D, the specific relationship with DPN still requires further investigation to be fully elucidated.

Concerning the observed relationship between African GA and DPN, this finding must be evaluated in the context of ethnicity, a subject of great interest worldwide that remains so far controversial, mainly due to different methods used to classify ethnicity [31, 43]. In the present study, we did not observe a difference among individuals with or without DPN according to self‐reported color‐race. However, a previous study performed by our group showed that self‐reported skin‐color can result in misclassifications, mainly due to the great miscegenation observed in the Brazilian population [12, 13]. Therefore, the availability of more specific methodologies such as GA can be more enlightening regarding the relationship between ethnicity and DPN. Our data showed that among the evaluated sociodemographic data, African GA had the highest association with DPN even after adjustments for socioeconomic status.

However, it is important to highlight that some SDOH are not included in the official socioeconomic assessment of the Brazilian population, such as neighborhood environment, food insecurity, access to health care, and stress, while some of these factors have been linked to DPN [9]. Nevertheless, it is important to emphasize that the influence of SDOH is not specific for diabetes, and it is very commonly associated with other diseases [44].

A recent cohort study that investigated mortality data in a multiethnic cohort in United States showed that the most important predictor of mortality were SDOH even after adjusting for African GA. Otherwise, the Brazilian population has a lower African GA (~20%) than African‐Americans (~75%) which results in a great different population stratification and in difficulties for comparing both studies [45]. In contrast, mortality risk among prostate cancer survivors was higher among Black vs. White and among individuals with higher African GA vs. European GA [46].

For instance, in the BrazDiab1SG, an increased African GA was associated with proliferative DR [47], metabolic syndrome [48] and a tendency to be associated with DKD [49], without an association with glycemic control [50]. Although the present study was not able to cover in detail the relationship between African GA and DPN, it should be noted that the increase in African GA was associated with an increase in the transcription of genes related to innate immune response, cytokine production, and bactericidal activity in an experimental model of infected macrophages with several live bacterial pathogens obtained from individuals that self‐identified as African‐Americans or European‐Americans [51]. These findings point to a significant ancestry‐related difference in immune responses, especially regarding the susceptibility to inflammation, which is an important diabetes‐related chronic complications driver [52]. The consistent association of African GA with the majority of diabetes‐related chronic complications observed and the present study suggests that African GA may play a broader role in modulating an individual's susceptibility to those complications through mediating immune response, inflammation, and other pathological mechanisms such as oxidative stress and endothelial dysfunction [52].

It is still unclear whether the relationship among GA and some diseases reflects inherent biological susceptibilities, such as ancestry‐specific genetic variants influencing immune activation, or whether GA may serve as a proxy for unmeasured SODH. However, to date, it has not been possible to establish a cutoff point of African GA that would be decisive in establishing its influence on diabetes‐related chronic complications, including DPN.

It is important to consider that around 34% of individuals with DPN may develop painful neuropathy and also have a higher risk of amputation [53]. Although we did not evaluate this clinical presentation separately, in the present cohort, individuals with DPN had a higher frequency of amputations. In general, individuals with T1D and diabetes‐related chronic complications, including DPN, had lower HRQoL as observed in the present study, associated with increased disability [54].

The main strength of our study is a very representative sample, as all five geographical Brazilian regions included participants that were characterized by different ethnic, cultural, socioeconomic, and genetic backgrounds. Also, a uniform and standardized recruitment protocol was applied in all centers. Lastly, to the best of our knowledge, this was the first multicenter study searching for a national prevalence of DPN in individuals with T1D in Brazil and also in South America.

Finally, our study had also limitations that must be addressed. First, our study had a cross‐sectional design that does not allow us to establish a causality association between the analyzed variables and DPN. Second, any type of hypoglycemia was self‐reported. Third, 495 patients were excluded from logistic regression analysis because they had two urine samples with divergent albuminuria results, making it impossible to classify them as having DKD, which probably underestimated DKD prevalence. Fourth, we selected secondary and tertiary health care clinics to participate in our study since very few patients with T1D in Brazil are followed in primary health care settings. Fifth, we used a 46 AIM‐INDEL panel and Structure software that, although being accepted and validated approaches for GA estimation, provide low‐resolution estimation compared to genome‐wide SNP arrays. However, in a recent paper evaluating the whole genome sequence data of healthy Brazilian individuals, the proportion of European, African, and Native Amerindian GA was similar to that observed in the present study, even considering that both populations were different regarding health status (individuals with diabetes vs. healthy controls) [13].

Lastly, similar to other epidemiological studies [2] we used only clinical criteria to define T1D presence of classic clinical presentation at diagnosis, such as polyuria, weight loss, polydipsia, and the need for continuous insulin use since then.

## 6 Conclusion

In conclusion, our study showed that individuals with T1D of a high ethnically admixed population had a relevant DPN prevalence. DPN was associated with several comorbidities and diabetes‐related complications. It is remarkable that they were young, had lower HRQoL, and some had been submitted to amputations, implying a high lifetime disease cost. The above‐mentioned facts shall drive future research areas concerning early detection and prevention of DPN. The association with the emerging risk factor African GA was very interesting but still deserves further studies involving other admixed populations such as the Brazilian.

## Author Contributions

M.B.G., L.R.M.T., C.H.A., H.C.P., C.A.N., D.S., L.L.F., L.C.P. writing and manuscript review. C.A.N. conceptualization. L.R.M.T., H.C.P., D.S., L.L.F., L.C.P. methodology. M.B.G. conceptualization, funding acquisition, project administration. D.S., L.L.F., L.C.P. genetic analysis. All the authors have approved the final version of the manuscript.

## Conflicts of Interest

The authors declare no conflicts of interest.

## Supporting information


**Supporting Information Table 1.** Brazilian Type 1 Diabetes Study Group (BrazDiab1SG).

## Data Availability

The data that support the findings of this study are available from the corresponding author upon reasonable request.

## References

[1] R. Pop‐Busui , A. J. M. Boulton , E. L. Feldman , et al., “Diabetic Neuropathy: A Position Statement by the American Diabetes Association,” Diabetes Care 40 (2017): 136–154. 27999003 10.2337/dc16-2042 PMC6977405

[2] K. R. Mizokami‐Stout , Z. Li , N. C. Foster , et al., “The Contemporary Prevalence of Diabetic Neuropathy in Type 1 Diabetes: Findings From the T1D Exchange,” Diabetes Care 43 (2020): 806–812. 32029635 10.2337/dc19-1583 PMC7085805

[3] D. Ziegler , C. Herder , and N. Papanas , “Neuropathy in Prediabetes,” Diabetes, Metabolic Syndrome and Obesity 39 (2023): e3693, 10.1002/dmrr.3693. 37470302

[4] B. C. Callaghan , R. Xia , M. Banerjee , et al., “Metabolic Syndrome Components Are Associated With Symptomatic Polyneuropathy Independent of Glycemic Status,” Diabetes Care 39, no. 5 (2016): 801–807. 26965720 10.2337/dc16-0081 PMC4839175

[5] B. C. Callaghan , L. Gao , Y. Li , et al., “Diabetes and Obesity Are the Main Metabolic Drivers of Peripheral Neuropathy,” Annals of Clinical Translational Neurology 5 (2018): 397–405. 29687018 10.1002/acn3.531 PMC5899909

[6] B. H. Braffett , R. A. Gubitosi‐Klug , J. W. Albers , et al., “Risk Factors for Diabetic Peripheral Neuropathy and Cardiovascular Autonomic Neuropathy in the Diabetes Control and Complications Trial/Epidemiology of Diabetes Interventions and Complications (DCCT/EDIC) Study,” Diabetes 69 (2020): 1000–1010. 32051148 10.2337/db19-1046 PMC7171957

[7] S. Tesfaye , A. J. M. Boulton , P. J. Dyck , et al., “Diabetic Neuropathies: Update on Definitions, Diagnostic Criteria, Estimation of Severity, and Treatments,” Diabetes Care 33 (2010): 2285–2293. 20876709 10.2337/dc10-1303 PMC2945176

[8] S. Tesfaye , N. Chaturvedi , S. E. Eaton , et al., “Vascular Risk Factors and Diabetic Neuropathy,” New England Journal of Medicine 352, no. 4 (2005): 341–350, 10.1056/NEJMoa032782. 15673800

[9] A. Jeyam , S. J. McGurnagha , L. A. K. Blackbourn , et al., “Diabetic Neuropathy Is a Substantial Burden in People With Type 1 Diabetes and Is Strongly Associated With Socioeconomic Disadvantage: A Population‐Representative Study From Scotland,” Diabetes Care 43 (2020): 734–742. 31974100 10.2337/dc19-1582

[10] M. Blankenburg , N. Kraemer , G. Hirschfeld , et al., “Childhood Diabetic Neuropathy: Functional Impairment and Non‐Invasive Screening Assessment,” Diabetic Medicine 29 (2012): 1425–1432. 22507184 10.1111/j.1464-5491.2012.03685.x

[11] T. Lrm , H. C. Pedrosa , C. H. Aguiar , et al., “Prevalence of Cardiovascular Autonomic Neuropathy in an Admixed Population of Patients With Type 1 Diabetes. Lessons From a Pioneer Multicenter Study in Brazil,” Primary Care Diabetes 18 (2024): 539–546, 10.1016/j.pcd.2024.08.001. 39152087

[12] M. B. Gomes , A. B. Gabrielli , D. C. Santos , et al., “Self‐Reported Color‐Race and Genomic Ancestry in an Admixed Population: A Contribution of a Nationwide Survey in Patients With Type 1 Diabetes in Brazil,” Diabetes Research and Clinical Practice 140 (2018): 245–252. 29574106 10.1016/j.diabres.2018.03.021

[13] K. Nunes , M. A. C. E. Silva , M. R. Rodrigues , et al., “Admixture's Impact on Brazilian Population Evolution and Health,” Science 388 (2025): eadl3564, 10.1126/science.adl3564. 40373151

[14] J. M. Galanter , J. C. Fernandez‐Lopez , C. R. Gignoux , et al., “Development of a Panel of Genome‐Wide Ancestry Informative Markers to Study Admixture Throughout the Americas,” PLoS Genetics 8, no. 3 (2012): e1002554, 10.1371/journal.pgen.1002554. 22412386 PMC3297575

[15] M. B. Gomes and C. A. Negrato , “Adherence to Insulin Therapeutic Regimens in Patients With Type 1 Diabetes. A Nationwide Survey in Brazil,” Diabetes Research and Clinical Practice 120 (2016): 47–55. 27513598 10.1016/j.diabres.2016.07.011

[16] IBGE , Censo 2010 [Internet], https://censo2010.ibge.gov.br/.

[17] N. A. ElSayed , G. Aleppo , V. R. Aroda , et al., “10. Cardiovascular Disease and Risk Management: Standards of Care in Diabetes—2023,” Diabetes Care 46, no. S1 (2023): S158–S190. 36507632 10.2337/dc23-S010 PMC9810475

[18] N. A. ElSayed , G. Aleppo , V. R. Aroda , et al., “6. Glycemic Targets: Standards of Care in Diabetes—2023,” Diabetes Care 46, no. S1 (2023): S97–S110. 36507646 10.2337/dc23-S006 PMC9810469

[19] W. Greiner , T. Weijnen , M. Nieuwenhuizen , et al., “A Single European Currency for EQ‐5D Health States. Results From a Six‐Country Study,” European Journal of Health Economics 4, no. 3 (2003): 222–231. 10.1007/s10198-003-0182-5 15609189

[20] I. H. de Boer , M. L. Caramori , J. C. N. Chan , et al., “KDIGO 2020 Clinical Practice Guideline for Diabetes Management in Chronic Kidney Disease,” Kidney International 98, no. 4 (2020): S1–S115. 32998798 10.1016/j.kint.2020.06.019

[21] G. J. Schwartz and D. F. Work , “Measurement and Estimation of GFR in Children and Adolescents,” Clinical Journal of the American Society of Nephrology 4, no. 11 (2009): 1832–1843. 19820136 10.2215/CJN.01640309

[22] C. P. Wilkinson , F. L. Ferris , R. E. Klein , et al., “Proposed International Clinical Diabetic Retinopathy and Diabetic Macular Edema Disease Severity Scales,” Ophthalmology 110, no. 9 (2003): 1677–1682. 13129861 10.1016/S0161-6420(03)00475-5

[23] R. O. Moreira , A. P. Castro , M. Papelbaum , et al., “Tradução Para o Português e Avaliação da Confiabilidade de Umaescala Para Diagnóstico da Polineuropatia Distal Diabética. Translation Into Portuguese and Assessment of the Reliability of a Scale for the Diagnosis of Diabetic Distal Polyneuropathy,” Arquivos Brasileiros de Endocrinologia e Metabologia 49, no. 6 (2005): 944–950, 10.1590/S0004-27302005000600014. 16544018

[24] M. J. Young , A. J. M. Boulton , A. F. MacLeod , D. R. Williams , and P. H. Sonksen , “A Multicentre Study of the Prevalence of Diabetic Peripheral Neuropathy in the United Kingdom Hospital Clinic Population,” Diabetologia 36 (1993): 150–154, 10.1007/BF00400697. 8458529

[25] H. C. Pedrosa , S. F. Tavares , M. A. C. Saigg , et al., “Programa Passo a Passo – Fichasclínicas,” in Neuropatias e pé Diabético, ed. H. C. Pedrosa , L. Vilar , and A. J. M. Boulton (AC Farmacêutica, 2014), 297–307.

[26] Censo 2000 , IBGE [Internet], https://www.ibge.gov.br/estatisticas/sociais/administracao‐publica‐e‐participacaopolitica/9663‐censo‐demografico‐2000.html?=&t=destaques.

[27] D. A. Malerbi and L. J. Franco , “Multicenter Study of the Prevalence of Diabetes Mellitus and Impaired Glucose Tolerance in the Urban Brazilian Population Aged 30‐69 Yr. The Brazilian Cooperative Group on the Study of Diabetes Prevalence,” Diabetes Care 15, no. 11 (1992): 1509–1516. 1468278 10.2337/diacare.15.11.1509

[28] CritérioBrasil ‐ ABEP [Internet], https://www.abep.org/criterio‐brasil.

[29] Instituto Nacional do Seguro Social – INSS , INSS [Internet], https://www.gov.br/inss/pt‐br/pagina‐inicial.

[30] R. Pereira , C. Phillips , N. Pinto , et al., “Straightforward Inference of Ancestry and Admixture Proportions Through Ancestry‐Informative Insertion Deletion Multiplexing,” PLoS One 7 (2012): e2968. 10.1371/journal.pone.0029684 PMC3260179 22272242

[31] J. Sun , Y. Wang , X. Zhang , S. Zhu , and H. He , “Prevalence of Peripheral Neuropathy in Patients With Diabetes: A Systematic Review and Meta‐Analysis,” Primary Care Diabetes 14, no. 5 (2020): 435–444, 10.1016/j.pcd.2019.12.005. 31917119

[32] C. Politi , C. Ciccacci , C. D'Amato , G. Novelli , P. Borgiani , and V. Spallone , “Recent Advances in Exploring the Genetic Susceptibility to Diabetic Neuropathy,” Diabetes Research and Clinical Practice 120 (2016): 198–208, 10.1016/j.diabres.2016.08.006. 27596057

[33] Q. Pan , Q. Li , W. Deng , et al., “Prevalence of and Risk Factors for Peripheral Neuropathy in Chinese Patients With Diabetes: A Multicenter Cross‐Sectional Study,” Frontiers in Endocrinology 9 (2018): 617, 10.3389/fendo.2018.00617. 30455667 PMC6230581

[34] C. Deng , Y. Xie , J. Li , et al., “Care, Control and Complications of Hospitalised Patients With Type 1 Diabetes in China: A Nationwide‐Based Registry Study,” Diabetes/Metabolism Research and Reviews 40, no. 3 (2024): e3796, 10.1002/dmrr.3796. 38529788

[35] M. Yovera‐Aldana , V. Velásquez‐Rimachi , A. Huerta‐Rosario , et al., “Prevalence and Incidence of Diabetic Peripheral Neuropathy in Latin America and the Caribbean: A Systematic Review and Meta‐Analysis,” PLoS One 16, no. 5 (2021): e0251642, 10.1371/journal.pone.0251642. 33984049 PMC8118539

[36] S. Balducci , G. Iacobellis , L. Parisi , et al., “Exercise Training Can Modify the Natural History of Diabetic Peripheral Neuropathy,” Journal of Diabetes and Its Complications 20, no. 4 (2006): 216–223. 16798472 10.1016/j.jdiacomp.2005.07.005

[37] S. T. Andersen , H. Wiggers , B. Hansen , E. T. Vestergaard , J. M. Bruun , and P. L. Kristensen , “Intensive Multifactorial Treatment and Cardiovascular Autonomic Neuropathy in Type 2 Diabetes: Results From the Randomized ADDITION‐Denmark Trial,” Diabetes Care 41, no. 3 (2018): 592–598.

[38] M. B. Gomes , M. H. Pizarro , L. H. Muniz , et al., “Prevalence of Chronic Kidney Disease in an Admixed Population of Patients With Type 1 Diabetes. A Multicenter Study in Brazil,” Diabetes Research and Clinical Practice 170 (2020): 108490. 33010359 10.1016/j.diabres.2020.108490

[39] L. G. N. Melo , P. H. Morales , K. R. G. Drummond , et al., “Current Epidemiology of Diabetic Retinopathy in Patients With Type 1 Diabetes: A National Multicenter Study in Brazil,” BMC Public Health 18, no. 1 (2018): 989. 30089461 10.1186/s12889-018-5859-x PMC6083618

[40] M. H. Pizarro , D. C. Santos , B. S. V. Barros , L. G. N. de Melo , and M. B. Gomes , “Serum Uric Acid and Renal Function in Patients With Type 1 Diabetes: A Nationwide Study in Brazil,” Diabetology and Metabolic Syndrome 10 (2018): 22. 29568334 10.1186/s13098-018-0324-7 PMC5859721

[41] A. S. Matheus , E. Tibiriçá , P. B. da Silva , M. de Fátima Bevilácqua Matta , and M. B. Gomes , “Uric Acid Levels Are Associated With Microvascular Endothelial Dysfunction in Patients With Type 1 Diabetes,” Diabetic Medicine 28, no. 10 (2011): 1188–1193, 10.1111/j.1464-5491.2011.03349.x. 21658123

[42] A. Abraham , A. Breiner , C. Barnett , et al., “Uric Acid Levels Correlate With the Severity of Diabetic Sensorimotor Polyneuropathy,” Journal of the Neurological Sciences 379 (2017): 94–98, 10.1016/j.jns.2017.05.053. 28716288

[43] J. M. Sosenko , “The Prevalence of Diabetic Neuropathy According to Ethnicity,” Current Diabetes Reports 9, no. 6 (2009): 435–439, 10.1007/s11892-009-0071-0. 19954688

[44] F. Hill‐Briggs and S. L. Fitzpatrick , “Overview of Social Determinants of Health in the Development of Diabetes,” Diabetes Care 46, no. 9 (2023): 1590–1598, 10.2337/dci23-0001. 37354331

[45] H. S. Iyer , I. Cheng , C. Opara , et al., “African Genetic Ancestry, Structural and Social Determinants of Health, and Mortality in Black Adults,” JAMA Network Open 8, no. 5 (2025): e2510016, 10.1001/jamanetworkopen.2025.10016. 40358946 PMC12076178

[46] J. B. Vo , D. W. Brown , I. D. Buller , et al., “Associations of Self‐Identified Race and Ethnicity and Genetic Ancestry With Mortality Among Cancer Survivors,” Journal of the National Cancer Institute (2025): djaf066, 10.1093/jnci/djaf066. 40112084 PMC12597501

[47] D. C. Santos , L. G. N. de Melo , M. H. Pizarro , et al., “Genomic Ancestry as a Risk Factor for Diabetic Retinopathy in Patients With Type 1 Diabetes From an Admixed Population: A Nested Case‐Control Study in Brazil,” Acta Diabetologica 57, no. 8 (2020): 937–945. 32125531 10.1007/s00592-020-01498-5

[48] B. S. V. Barros , D. C. Santos , L. G. N. Melo , et al., “Genomic Ancestry and Metabolic Syndrome in Individuals With Type 1 Diabetes From an Admixed Population: A Multicentre, Cross‐Sectional Study in Brazil,” Diabetic Medicine 38, no. 2 (2021): e14400. 32918322 10.1111/dme.14400

[49] M. H. Pizarro , D. C. Santos , L. G. N. Melo , et al., “Influence of Genomic Ancestry and Self‐Reported Color‐Race in CKD in a Nationwide Admixed Sample of Brazilian Patients With Type 1 Diabetes,” Diabetes, Metabolic Syndrome and Obesity 12 (2019): 1831–1840. 10.2147/DMSO.S210585 PMC6748312 31571958

[50] M. B. Gomes , L. E. Calliari , D. C. Santos , et al., “Genomic Ancestry and Glycemic Control in Adolescents With Type 1 Diabetes: A Multicenter Study in Brazil,” Pediatric Diabetes 21, no. 5 (2020): 727–734, 10.1111/pedi.13031. 32335987

[51] Y. Nédélec , J. Sanz , G. Baharian , et al., “Genetic Ancestry and Natural Selection Drive Population Differences in Immune Responses to Pathogens,” Cell 167, no. 3 (2016): 657–669.e21, 10.1016/j.cell.2016.09.025. 27768889

[52] Z. Z. Chong and N. Souayah , “Crumbling Pathogenesis and Biomarkers for Diabetic Peripheral Neuropathy,” Biomedicine 13, no. 2 (2025): 413, 10.3390/biomedicines13020413. PMC11853266 40002826

[53] Y. Tao , H. Y. Zhang , C. MacGilchrist , E. Kirwan , and C. McIntosh , “Prevalence and Risk Factors of Painful Diabetic Neuropathy: A Systematic Review and Meta‐Analysis,” Diabetes Research and Clinical Practice 222 (2025): 112099, 10.1016/j.diabres.2025.112099. 40107621

[54] A. C. Braga de Souza , J. S. Felício , C. C. Koury , et al., “Health‐Related Quality of Life in People With Type 1 Diabetes Mellitus: Data From the Brazilian Type 1 Diabetes Study Group,” Health and Quality of Life Outcomes 13 (2015): 204. 26703221 10.1186/s12955-015-0396-0 PMC4690259

